# Uncertain imputation for time-series forecasting: Application to COVID-19 daily mortality prediction

**DOI:** 10.1371/journal.pdig.0000115

**Published:** 2022-10-25

**Authors:** Rayane Elimam, Nicolas Sutton-Charani, Stéphane Perrey, Jacky Montmain

**Affiliations:** EuroMov Digital Health in Motion, Univ Montpellier, IMT Mines Ales, Ales, France; UCSF: University of California San Francisco, UNITED STATES

## Abstract

The object of this study is to put forward uncertainty modeling associated with missing time series data imputation in a predictive context. We propose three imputation methods associated with uncertainty modeling. These methods are evaluated on a COVID-19 dataset out of which some values have been randomly removed. The dataset contains the numbers of daily COVID-19 confirmed diagnoses (“new cases”) and daily deaths (“new deaths”) recorded since the start of the pandemic up to July 2021. The considered task is to predict the number of new deaths 7 days in advance. The more values are missing, the higher the imputation impact is on the predictive performances. The Evidential *K*-Nearest Neighbors (E*K*NN) algorithm is used for its ability to take into account labels uncertainty. Experiments are provided to measure the benefits of the label uncertainty models. Results show the positive impact of uncertainty models on imputation performances, especially in a noisy context where the number of missing values is high.

## Author Summary

The methodological aim of this study was to take advantage of missing data chronology in the imputation process in order to handle missing time series data. The practical goal of COVID application was to study the link between the numbers of chronological COVID confirmed cases and death. To achieve these goals we proposed 3 imputation methods of missing time series data each of them associated with an uncertainty model. For the COVID number of death prediction task, we set up a non-linear regression modeling for the number of COVID deaths prediction from past deaths and confirmed cases data. This led us to extend the Evidential K-Nearest Neighbor method to regression problems and to assess the impact of uncertainty modeling within imputation process in regards to predictive task. Finally, we showed the superiority of the time-E*K*NN (TEKNN) in terms of predictive performances compared to the Last Observation Carried Forward (LOCF) and Centered Moving Average (CMA) methods. More globally, we showed the interest of modeling the uncertainty in the imputation process to better control the prediction error, especially during relative stable periods.

## 1 Introduction

With an increasing number of machine learning applications, data availability is becoming very important. Yet available datasets are often incomplete due to different measurement failures, especially when the data collection involves human participation. The treatment of missing values for predictive tasks has become an important issue giving rise to a wide range of research. Many methods have been proposed to handle missing values (average, omission, learning, etc.), one of the most popular being simply to exclude incomplete examples from the learning set, due to the incapacity to deal with missing values of most predictive models [1, 2]. That type of treatment remains undesirable with limited amounts of available data or in a chronological data structure.

The chosen method also depends on the nature of the missing values, which is often categorized in *Missing At Random* (MAR) for missing values that are dependent on observed values, *Not Missing At Random* (NMAR) missing values which depend on unobserved values and *Missing Completely At Random* (MCAR) missing values which are independent of observed or unobserved values [3, 4]. Those categories indicate why data are missing, an information to be taken into account in the imputation method [5].

Moreover, in a time-series forecasting context, missing values introduce irregular time stamps that contradict the most common hypothesis of standard time series methods. In terms of uncertainty, missing values can be interpreted as total ignorance or complete imprecision about the actual values. Some soft computing methods are designed to handle data uncertainty by modeling its degree [6–8]. In such frameworks, ignorance corresponds to the highest level of uncertainty and therefore missing values can be incorporated in models that take into account the uncertainty level. In this study our aim is to predict COVID-19 daily deaths in an artificially noised dataset out of which some labels (number of new deaths) have been randomly removed, resulting in MCAR missing values since the missingness is not related to any observed or unobserved values. The benefits of associating uncertainty models to imputation methods are studied. We evaluate the predictive performance of the Evidential-*K* Nearest Neighbors algorithm once missing data are imputed with and without uncertainty models (in the latter case the imputed labels are considered as certain).

The structure of the dataset is adapted to time series forecasting. We propose a methodology to handle the uncertainty inherent to missing values imputation methods. Some theories allow representation of uncertainty in a broader way than classical probability theory. Missing values uncertainty can be handled in different frameworks, *e.g.* fuzzy sets [9], possibilities distribution [10], probability sets [11], belief functions [12, 13]. We chose the belief functions framework for its flexibility and relative simplicity and also because recognized machine learning algorithms based on that framework are available [14–17].

Beyond standard machine learning researches on missing data imputation methods [1, 2], some soft computing imputation methods have been proposed [18–20]. In [21], a method is proposed to categorize missing data and to remove noise with a kernel-based approach that enables classification within the belief function framework. The purpose of the method is to design an imputation strategy providing uncertainty *resistance*; however the method does not handle the uncertainty at the predictive level. In [22] the authors propose a method to minimize the classification errors due to uncertainty caused by missing values. Multiple precise missing values estimations are performed and the corresponding predictions are finally combined in predictive belief functions. In the context of information retrieval, Jousselme *et al.* proposed a missing values uncertainty representation [23]. Missing data are modeled as a belief function defined over the variables spaces. The method shows good performance for information retrieval task. As a matter of fact, none of those methods allows for the taking into account the uncertainty associated with imputation at the predictive level. In this study, we propose an approach to impute missing data in a chronological dataset and to model the resulting uncertainty in the belief functions framework. Finally an evidential classification model (E*K*NN) is extended to regression tasks in order to take into account the uncertainty associated with the imputation process.

The rest of this paper is organized as follows: first we present the main results of this study in Section 2, then we present our conclusion and perspectives in Section 3. All the details of our approach are given in Section 4 where we briefly recall the basis of the belief functions framework basis and the E*K*NN algorithm in the first subsection 4.1. After the presentation of the time series forecasting problem in an incomplete data context in the following subsection 4.2, three missing value imputation methods are proposed in subsection 4.3. In subsection 4.4 we present the uncertainty models associated with the previously introduced imputation methods; The uncertainty we are handling in this study is epistemic as we have no information about the missing label values. The chosen predictive model is Evidential-*K* Nearest Neighbors for its simplicity and its ability to deal with uncertain labels [14].

## 2 Results

**Fig 1:**
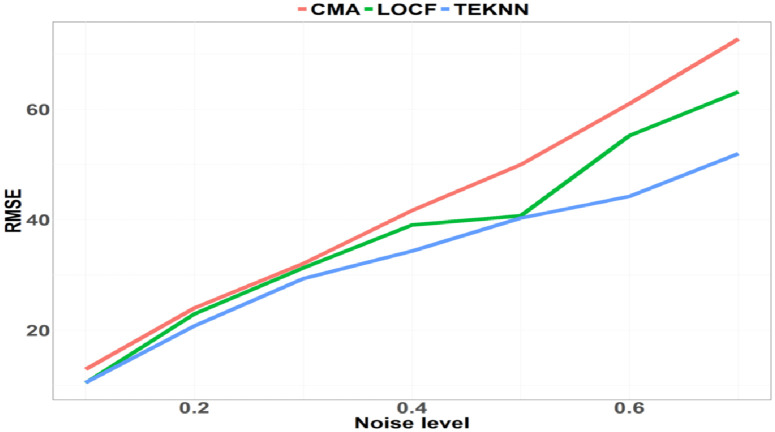
Imputation errors.

First, we observe in Fig 1 that the three imputation methods are comparable in terms of performance. The TEKNN approach seems to perform better than the LOCF and CMA methods and its superiority grows as the noise level increases. Except for a small noise level of 0.1, the TEKNN model seems to be the best imputation method.

**Fig 2:**
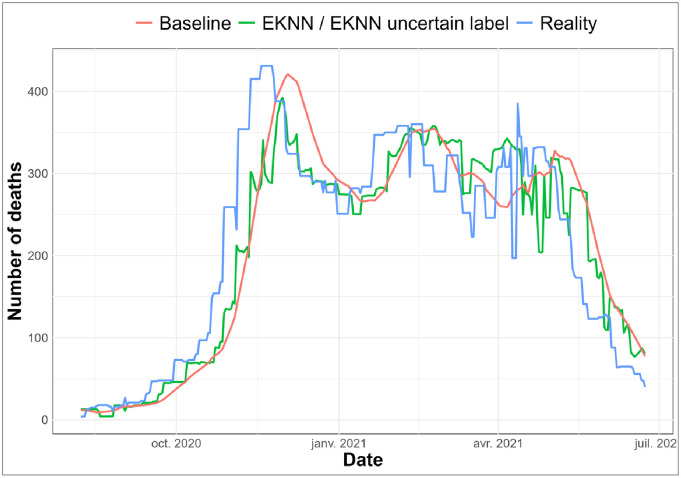
Predictive results on data imputed with time-E*K*NN imputation method, comparison with true labels: *K* = 1: *q* = 4: *v* = 0.

On the chronological evaluation with a noise level of 0 (Fig 2), we observe that the E*K*NN predictions with and without uncertainty models associated to label imputation blend together. This observation was expected as the data are not noised, *i.e.* there is no uncertainty associated with training labels. During the increasing and decreasing phases of the number of deaths, the E*K*NN seems to perform better than the baseline approach (blue and green curves are closer to the purple one than the red curve during those periods). During the periods of relative stability when the evolution of the pandemic slows down, there seems to be no significant differences between the considered approaches.

We note a small time shift between the real number of daily deaths and all predictive models, especially at the beginning of the wave. This is due to the fact that the model needs high number of deaths in the past to be able to predict high values in the future.

We observe a phase shift at the start of the wave, due to the fact that, before, there is no neighbor labelled with a high number of deaths, therefore the predicted values are under-estimated until we have data in the training set presenting a high number of deaths. We also note that the phase shift reduces thereafter.

**Fig 3:**
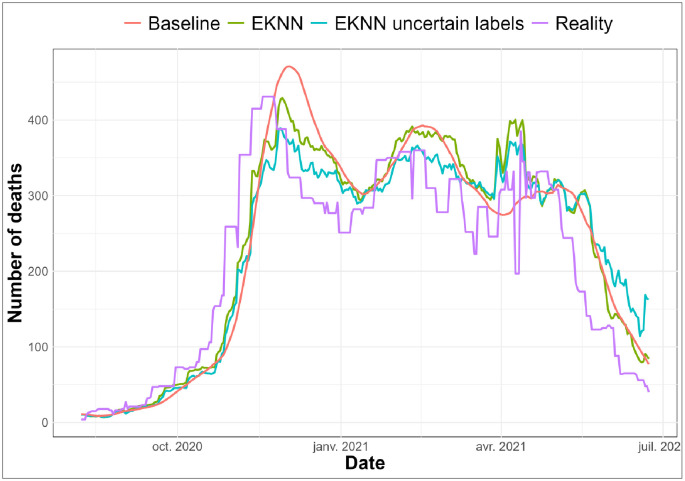
Predictive results on data imputed with time-E*K*NN imputation method, comparison with true labels: *K* = 1: *q* = 3—*v* = 0.3.

On Fig 3, we observe that, with time-E*K*NN imputations, the “E*K*NN uncertain labels” and the “E*K*NN” make predictions reaching quite similar performances with a slight superiority for the “E*K*NN” (without uncertain model) during the beginning and the end phases of the pandemic wave. During the relatively stable periods, the “E*K*NN” associated with an uncertain model performs better than without imputation uncertain modelling.

**Fig 4:**
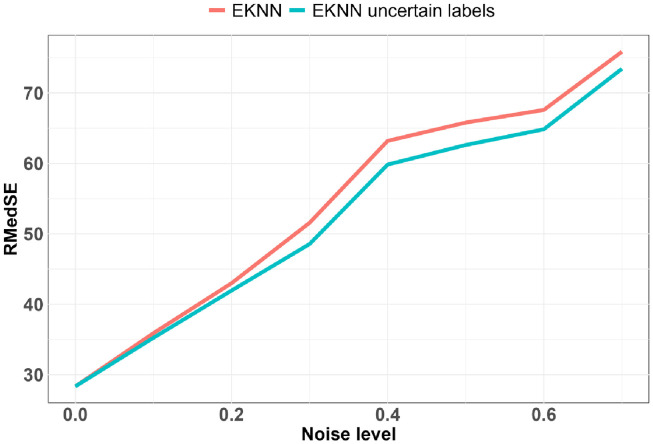
Predictive performances relative to noise levels for data imputed with LOCF imputation method: K = 10: z = 4.

**Fig 5:**
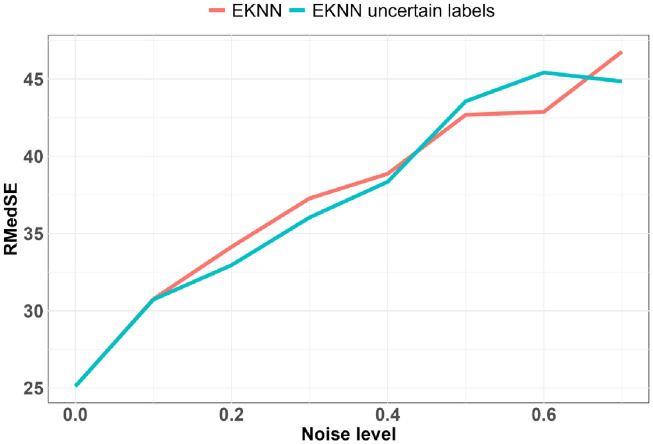
Predictive performances relative to noise levels for data imputed with time-E*K*NN imputation method: *K* = 1: *q* = 4.

**Fig 6:**
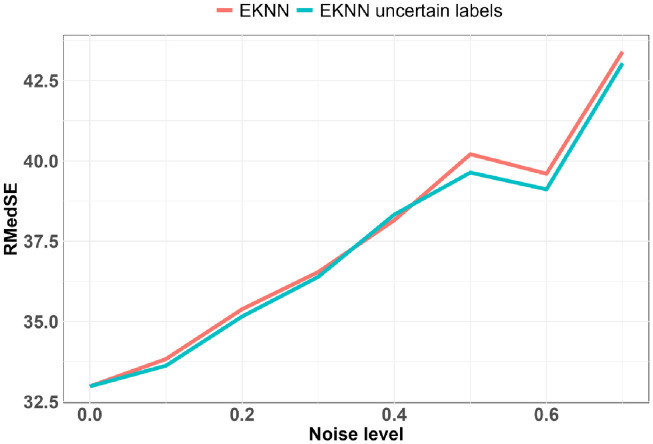
Predictive performances relative to noise levels for data imputed with CMA imputation method: *K* = 20: *q* = 4.

The best results with a noise level of zero were obtained with *K* = 1, and *q* = 4. We see on Fig 5 that thanks to the uncertainty model of the TE*K*NN imputation method we have a better predictive performance up to a high noise level. The superiority of the standard E*K*NN after a noise level of about 0.5 is due to the fact that a high missing value rate induces highly uncertain neighborhoods and thus very uncertain predictions, a large mass being attributed to ignorance. The pignistic transformation applied to the mass function output of the E*K*NN distributes the mass on the Ω space in a uniform way on all the singletons; if this mass is too high, the predictions tend to the center of the space. Except for *K* = 1, for all the other configurations *K* = {10, 20} and *q* = {1, 2, …, 7} the use of uncertainty models allows us to have better predictive performances (Figs 4, 5 and 6).

Redefining Ω at each iteration depending on the maximum number of deaths observed is a conservative way to proceed, but it allows both models to predict any “reasonable” unobserved value.

## 3 Conclusion

The aim of this study was to propose uncertainty models associated with missing chronological data imputation methods. The objective was the prediction of the number of daily COVID 19 deaths at a prediction horizon of 7 days with an artificially noised dataset. We proposed three imputation methods (LOCF,CMA,time-E*K*NN) that showed good imputation performances.

For our experiment we extended the E*K*NN methodology proposed in [14] to regression problems. We were able to compare the predictive performances of the “E*K*NN” and the “E*K*NN uncertain labels” with three imputation methods of comparable performances. The experiment showed the benefit of uncertainty modeling for chronological imputed values throughout several hyper-parameters configurations.

The use of incomplete past values (*x*_*t*_, *y*_*t*_)_*t*=*t*−*q*,…,*t*_ as features leads to uncertain feature values. A logical continuation of this work could be to use other predictive models than the E*K*NN, especially the ones that handle uncertain attributes during learning [17, 24, 25].

The problem of predicting COVID 19 daily deaths led us to a numerical regression problem, therefore the time based uncertainty model is not adapted to classification. It would be interesting to extend it to classification in a categorical time series context. We also know from health experts that the number of new COVID 19 cases is not a good indicator for predicting deaths, therefore it would be interesting to weigh the importance of the attributes in the *K* nearest neighbors computing [26].

Another perspective could be to compare the predictive performance we can get with soft predictive models that handle missing values without any need of imputation.

Additionally, there are some algorithms like E*K*NN that use this framework. The theory of belief functions permits us to have the enhancement of uncertainty modeling as a perspective, for example by using imputation with intervals instead of precise values.

## 4 Materials and methods

### 4.1 Background

In this section we expose the basics of belief functions theory, also known as Dempster-Shafer or evidence theory [12, 13] and we detail the Evidential *K*-Nearest Neighbors algorithm [14].

#### 4.1.1 Belief functions

Let Ω = {*ω*_1_, *ω*_2_, …, *ω*_*H*_} be the so-called frame of discernment, *i.e.* the universe of possible outcomes or hypotheses. The mass function *m* represents our degree of knowledge about all subsets of Ω, *i.e.* about the powerset 2^Ω^ of Ω. The elements *A* ⊆ Ω such as *m*(*A*) > 0 are called focal elements and their weights sum to 1:

$$\begin{array}{c} \sum_{A\subseteq \Omega }m\left(A\right)=1 \end{array}$$ (1)

The quantity *m*(Ω) represents the degree of ignorance. From the mass function *m*, different uncertainty measures can be computed such as the *belief* and *plausibility* functions defined in Eqs (2) and (3) which can be interpreted as lower and upper “bounds of probability”.

$$\begin{array}{c} Bel\left(A\right)=\sum_{B\subseteq A}m\left(B\right) \end{array}$$ (2)

$$\begin{array}{c} Pl\left(A\right)=\sum_{B\cap A\neq Ø}m\left(B\right) \end{array}$$ (3)

Different mass functions can represent different sources of information. At the decision level, it may be necessary to combine them into a single mass function expressing all the knowledge we can infer from these sources.

**Mass combination**

There are multiple methods of information fusion through mass combination rules [27, 28]. One of the most famous is the Dempster’s conjunctive rule of combination ⊕ [12] (see Eq (4)):

$$\begin{array}{c} \left(m_{1}⊕m_{2}\right)\left(A\right)=\frac{1}{1-\kappa }\sum_{B\cap C=A}m_{1}\left(B\right)m_{2}\left(C\right) \end{array}$$ (4)

where $\kappa =\sum_{B\cap C=\emptyset }m_{1}\left(B\right)m_{2}\left(C\right)$ is the degree of *conflict* between sources *m*_1_ and *m*_2_.

The main idea of this rule is to consider all sources reliable. After the combination, we get a new mass function that can be used at the decision making level.

**Decision making**

In cases where the degree of ignorance *m*(Ω) is too important, some authors recommend rejecting decision making [14]. Otherwise, the choice of uncertainty measure to make a decision presents a dilemma [29].

For instance, depending on the application goal and the chosen strategy in terms of conservatism, any uncertainty measure lying between the *belief* (2) and the *plausibility* (3) measures can be used. However, those measures are not additive, *i.e.* we do not have *Bel*(*A* ∪ *B*) ≠ *Bel*(*A*) + *Bel*(*B*) ∀*A*, *B* ∈ Ω such as *A* ∩ *B* = ∅ (same thing for the *Pl* function). For that reason many data science tools are incompatible with those *soft* uncertainty measures since most of them have been developed within the standard probability framework.

For pragmatic reasons many researchers choose to project the information content of mass functions into the standard probability framework [14, 17]. The *Transferable Belief Model* was proposed by Smets [29, 30] where the *pignistic* transformation allows to convert mass functions into standard probability distributions. Despite known drawbacks [31], ignorance degrees are projected into uniform distributions. The pignistic transform defined in Eq (5) remains a natural solution for computing probability distributions from mass functions that mainly relies on uniform ignorance modeling.

In the machine learning context many classifiers make soft predictions expressed in more complex spaces than the standard probability one [14, 32]. When the learning data are uncertain (evidential), in order to get *handy* predictions some authors [17] have proposed to maximize the evidential extension of the likelihood function [14] in order to estimate probability distributions. When the evidential likelihood maximization is not straightforward, the Evidential Expectation Maximization (E^2^M) algorithm can be used. However the iterative nature of the E^2^M algorithm can lead to high levels of complexity. For the sake of simplicity, the pignistic transform is preferred in this study.

$$\begin{array}{c} BetP\left(\omega \right)=\frac{1}{1-m(\emptyset )}\sum_{A⊇\omega }\frac{m(A)}{\left|A\right|}\;\;\;\forall \omega \in \Omega \end{array}$$ (5)

#### 4.1.2 Evidential *K*Nearest Neighbors—EKNN

The E*K*NN extends *K*-Nearest Neighbors algorithm to the belief functions framework [14] and is based on Dempster’s conjunctive rule of combination. Let (*x*, *y*) = (*x*_*i*_, *y*_*i*_)_*i*=1,…,*n*_ be a training set and Ω = {*ω*_1_, *ω*_2_, …., *ω*_*H*_} the frame of discernment of the class label *Y*. Let *x*_*s*_ be a new observation to classify. The first step is to compute the distances between *x*_*s*_ and all training examples *x*_*i*_ to get the set of the *K* “nearest” neighbors of *x*_*s*_. In the E*K*NN approach, each neighbor is considered as a source of information. For each neighbor *x*_*i*_ labelled with {*ω*_*l*_}, a mass function *m*_*s*,*i*_ is computed:

$$\{\begin{array}{c} \begin{array}{ccc} m_{s,i}\left(\left\{\omega_{l}\right\}\right) & = & \alpha_{0}\times exp\left(-\gamma_{l}^{2}\times d_{s,i}^{\lambda }\right)\\ m_{s,i}\left(\Omega \right) & = & 1-m_{s,i}\left(\left\{\omega_{l}\right\}\right) \end{array} \end{array}$$ (6)

The quantity *m*_*s*,*i*_({*ω*_*l*_}) represents the mass assigned to the label *ω*_*l*_ by neighbor *x*_*i*_ to *x*_*s*_. The parameters *α*_0_, *γ*_*l*_ can be estimated with classical optimization procedure as gradient descent. The parameter *γ*_*l*_ > 0 relates to the label *ω*_*l*_, in [14] the author recommends to set the parameter *α*_0_ (which prevents dogmatic mass functions) to 0.95, *d*_*s*,*i*_ stands for the euclidean distance between *x*_*s*_ and its neighbor *x*_*i*_ and λ ∈ {1, 2, 3, …} is a parameter that penalizes the farthest neighbors.

Once all the masses *m*_*s*,*i*_ have been computed, they are combined with Dempster’s rule of combination into a final mass $m_{s}=m_{s,i_{1}}⊕...⊕m_{s,i_{K}}$ where $m_{s,i_{1}},...,m_{s,i_{K}}$ represent the knowledge associated with the *K* nearest neighbors of *x*_*s*_. Finally, decision can be made according to *m*_*s*_, the approach chosen in [14] is to predict the label corresponding to the maximum of belief.

In the case of uncertain or imperfect labels modeled by mass functions, *i.e.* the learning set is now ${\left(x_{i},m_{y_{i}}\right)}_{i=1,...,n}$, for each neighbor *x*_*i*_ we have a mass function $m_{y_{i}}$ on the label variable *Y*. In [14], the author proposes to discount the mass functions of all neighbors with the uncertainty level of their labels. In Eq (6), the term corresponding to the uncertainty level of the labels $m_{y_{i}}$ is added which results in Eq (7).

$$\{\begin{array}{c} \begin{array}{ccc} m_{s,i}\left(A\right) & = & \alpha_{0}\times exp\left(-\gamma_{A}^{2}\times d_{s,i}^{\lambda }\right)\times m_{y_{i}}\left(A\right)\;\;\;\forall A\subseteq \Omega \\ m_{s,i}\left(\Omega \right) & = & 1-\sum m_{s,i}\left(A\right) \end{array} \end{array}$$ (7)

In this study we deal with a regression problem since the number of COVID-19 daily deaths is numerical. We therefore extend the original E*K*NN model, that was initially designed for classification problems, to discrete regression tasks. To do so we removed all *γ*_*l*_ parameters (defined relatively to categorical class labels) from Eqs (6) and (7), which results in Eqs (8) and (9).

E*K*NN uncertainty model for regression:

$$\begin{array}{ccc} \text{precise}\;\text{labels} & : & \left\{ \begin{array}{ccc} m_{s,i}\left(\left\{\omega_{l}\right\}\right) & = & \alpha_{0}\times exp\left(-d_{s,i}^{\lambda }\right)\\ m_{s,i}\left(\Omega \right) & = & 1-m_{s,i}\left(\left\{\omega_{l}\right\}\right) \end{array}\right. \end{array}$$ (8)

$$\begin{array}{ccc} \text{uncertain}\;\text{labels} & : & \left\{ \begin{array}{ccc} m_{s,i}\left(\left\{\omega_{l}\right\}\right) & = & \left(\alpha_{0}\times exp\left(-d_{s,i}^{\lambda }\right)\right)\times m_{i}\left(\left\{\omega_{l}\right\}\right)\\ m_{s,i}\left(\Omega \right) & = & 1-m_{s,i}\left(\left\{\omega_{l}\right\}\right) \end{array}\right. \end{array}$$ (9)

The implementation of this extension of the E*K*NN algorithm to regression is available on our github (https://github.com/lgi2p/evidential_imputation).

For decision making (*i.e.* prediction) we used the pignistic transform *BetP*_*s*_ of *m*_*s*_ in order to predict the pignistic expectation.

The predicted label of a new observation *x*_*s*_ is:

$$\begin{array}{c} E_{BetP_{s}}\left[Y\right]=\sum_{\omega \in \Omega }BetP_{s}\left(\left\{\omega \right\}\right)\times \omega \end{array}$$ (10)

### 4.2 Formalism

In this section we present the formalism of both the regression problem and the missing value imputation task.

#### 4.2.1 Predictive problem

Let *D* = (*x*_*t*_, *y*_*t*_)_*t*=0,…,*T*_ be a dataset where $x_{t}\in \Omega_{X}\subseteq N$ and $y_{t}\in \Omega_{Y}\subseteq N$ are respectively the feature and label values at time *t*. We suppose that some label values are missing, *i.e.* some *y*_*t*_ values are not known. The aim of the regression task is to approximate a function *f* mapping current and past features values to future labels:

$$\begin{array}{c} y_{t}=f\left(y_{t-h},...,y_{t-(h+q)},x_{t-h},...,x_{t-(h+q)}\right) \end{array}$$ (11)

where *h* is the prediction horizon, *q* a number of past features and label values to consider. This regression modelling implies that, at any timestamp *t*, the number of deaths *y*_*t*_ can be predicted from the concatenation of the sets of previous number of death (*y*_*t*−*h*_, …, *y*_*t*−(*h*+*q*)_) and of previous number of cases (*x*_*t*−*h*_, …, *x*_*t*−(*h*+*q*)_).

Predicting deaths from data restricted to past number of death and cases is not usual in COVID forecasting works since some useful variables as the *basic reproduction number* *R*_0_, hospital entries, exits and intensive care daily numbers, state health measures (confinement, etc) are generally used for predicting future deaths. In our case we chose to restrict to deaths and cases variables as most of the other previously stated variables are usually incomplete in public datasets. Indeed, our work is based on the E*K*NN model which can deal with uncertain labels but not uncertain features (in its initial form).

Moreover, restricting ourselves to only 2 types of data (deaths and cases) makes experiments easier to run. Nevertheless, all this work can be easily extended to high-dimensionality features provided they are not incomplete in dataset. It is worth mentioning that some work has extended the E*K*NN model to uncertain features by computing distances between examples based on Jousselme distance which can be computed between belief function and thus between uncertain features [24].

Since some of *D*’s values are missing, the imputation process has to occur upstream. In Eq (10) past labels *y*_*t*_ are inputs of the function as historic features. Therefore removing incomplete examples introduces irregular timestamps in the data, which is inconsistent with the regularity hypothesis of most time series treatments.

#### 4.2.2 Imputation problem

Let us consider $y_{p_{1}}...y_{p_{U}}$ the *U* known previous label values with *p*_*U*_ < … < *p*_1_ before a missing label *y*_*t*_ and $y_{n_{1}}...y_{n_{R}}$ with *n*_1_ < … < *n*_*R*_ the next *N* known values, *U* and *R* are hyper-parameters that have to be tuned.

**Fig 7:**
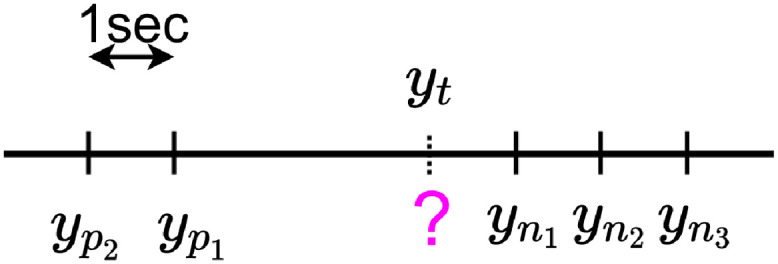
Chronological data imputation.

In the example presented in Fig 7 we have *P* = 2 and *N* = 3.

The aim of the imputation process is to compute or *impute* a value $\hat{y_{t}}$ for all missing *y*_*t*_.

### 4.3 Imputation methods

In this section we present three imputation methods to impute $\hat{y_{t}}$. The first one is the Last Observation Carried Forward (LOCF) method that replaces missing values with the last known value. The second one is the Centered Moving Average imputation (CMA) method that takes into account the dynamical nature of the data, and imputes missing values from the nearest future and past values. The last one is the “time-E*K*NN” (TEKNN) imputation method that applies the E*K*NN algorithm with a temporal distance to predict missing values, this method also takes into account the dynamical nature of the data as CMA method. The three imputation methods considered in this study are based on the use of those past and future label values.

#### 4.3.1 Last Observation Carried Forward (LOCF)

**Fig 8:**
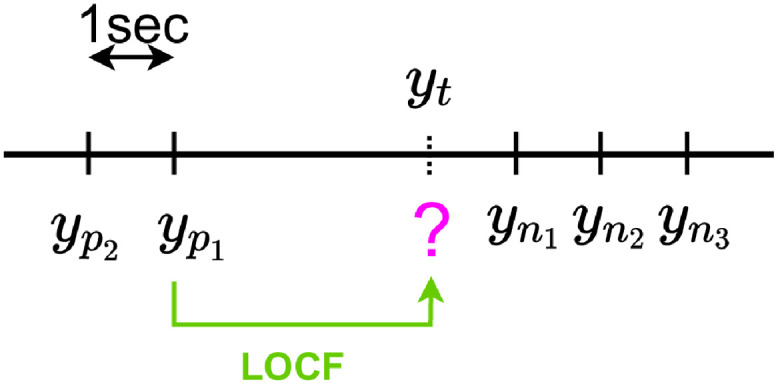
LOCF imputation.

Let *y*_*t*_ be a missing label value and $y_{p_{1}}$ the last known value. The LOCF imputation is simply $\hat{y_{t}}=y_{p_{1}}$ as illustrated in Fig 8.

#### 4.3.2 Centered Moving Average (CMA) imputation

**Fig 9:**
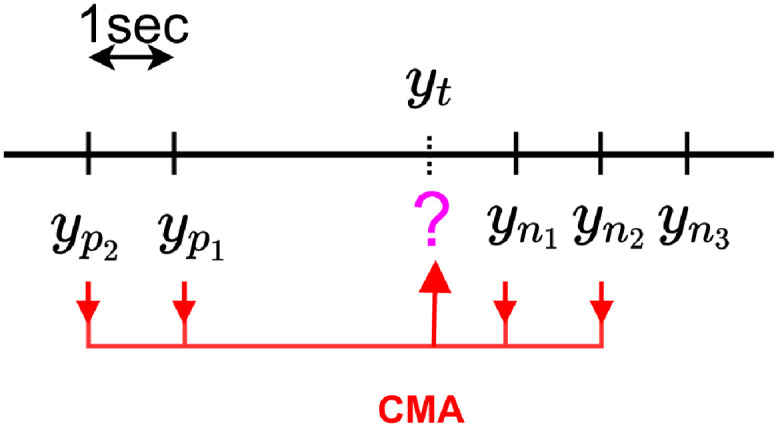
CMA imputation.

Here we preset a method taking into account the dynamical nature of the data. It is based on the intuition that labels close in time are likely to have close values. More simply, the idea of the CMA imputation method is to impute the missing *y*_*t*_ from the nearest known past and future labels (see Fig 9).

The past and future label values are averaged according to the duration between them and the missing label *y*_*t*_. Let $\delta_{t}^{p}=\left|t-p\right|$ and $\delta_{t}^{n}=\left|t-n\right|$ be respectively the time shifts between the time *t* of a missing label *y*_*t*_ and the time of the previous and next known label values, we have:

$$\begin{array}{c} \hat{y_{t}}=\sum_{u=1}^{U}Sim\left(t,p_{u}\right)\times y_{p_{u}}+\sum_{r=1}^{R}Sim\left(t,n_{r}\right)\times y_{n_{r}} \end{array}$$ (12)

with $\forall \left(t,U,R\right)\in \left\{0,...,T\right\}\times {N^{*}}^{2}:$

- $$\begin{array}{c} Sim\left(t,p_{u}\right)=\frac{sim(t,p_{u})}{\sum_{u=1}^{U}sim\left(t,p_{u}\right)+\sum_{r=1}^{R}sim\left(t,n_{r}\right)} \end{array}$$ (13)
- $$\begin{array}{c} sim\left(t,p_{u}\right)=1-\frac{\delta_{t}^{p_{u}}}{\sum_{u=1}^{U}\delta_{t}^{p_{u}}+\sum_{r=1}^{R}\delta_{t}^{n_{r}}} \end{array}$$ (14)

Eqs (13) and (14) define a normalized temporal similarity *Sim*(*t*, *p*_*u*_) between a measurement time *t* and one of the previous measurement times *p*_*u*_. Those similarities are used to weigh past label values in Eq (12). Note that these equations can be directly transposed to measure the similarity *Sim*(*t*, *n*_*r*_) between *t* and any next measurement time *n*_*r*_.

For the example of Fig 7, the CMA imputation with *U* = *R* = 2 leads to $\hat{y_{t}}=\frac{6}{30}\times y_{p_{2}}+\frac{7}{30}\times y_{p_{1}}+\frac{9}{30}\times y_{n_{1}}+\frac{8}{30}\times y_{n_{2}}$

#### 4.3.3 Time-E*K*NN (TEKNN) imputation

**Fig 10:**
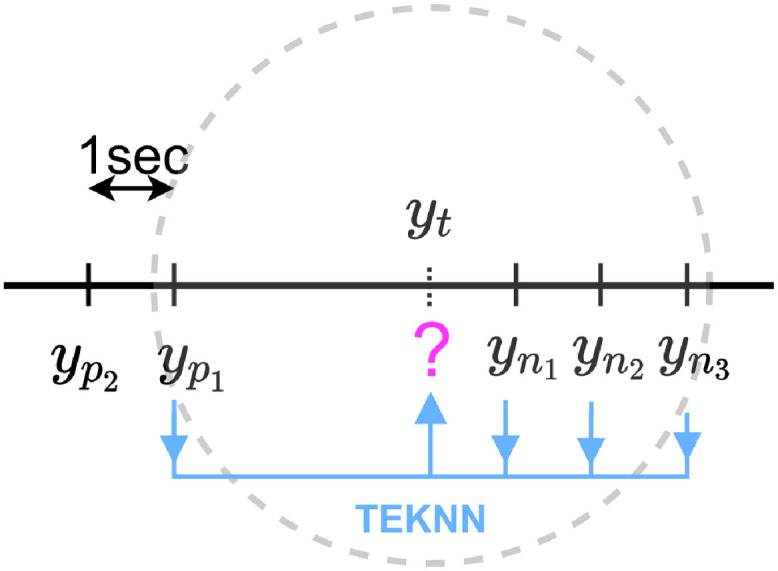
TE*K*NN imputation.

The idea behind this imputation approach is to use the E*K*NN regression model to predict the missing label values based on the complete examples, *i.e.* where label values are known, that are the closest in time. This method could be seen as a de-centered extension of the CMA approach where the points used for imputation are the closest regardless of their temporal disposition around the missing value (before and/or after) as in Fig 10. For this model too, the nearest neighbors on time have more weights on the imputed values ${\hat{y}}_{t}$ (see Eq (8)).

Regardless of the considered method, by nature the imputation of missing data involves some uncertainty associated with the imputed values. The next subsection proposes an uncertainty model for imputed time series data within the belief function framework.

### 4.4 Uncertainty modeling for imputation methods

In this subsection we present the uncertainty models associated with the 3 imputation methods described in the previous subsection. After uncertainty modeling, mass functions $m_{t}^{y}$ are assigned to each label *y*_*t*_ whether its value is missing or not. For known label values, categorical mass functions $m_{t}^{y}\left(\left\{y_{t}\right\}\right)=1$ are assigned. The imputation methods associated with the uncertainty model allow the conversion of a precise but incomplete (in terms of labels) dataset (*x*, *y*) into a complete evidential dataset ${\left(x_{t},m_{t}^{y}\right)}_{t=1,\ldots ,T}$.

Because of their dynamical nature, the LOCF and CMA imputation methods are associated with a time-based uncertainty model in the rest of this article. The idea behind this is that the closer in time the values used for the imputation of the missing labels are, the less uncertain the resulting imputed labels will be.

The uncertainty model of the time-E*K*NN imputation approach is the E*K*NN’s evidential output.

### 4.5 Time based model

Once missing labels *y*_*t*_ have been imputed with the LOCF or CMA method into precise computed values $\hat{y_{t}}$, this paragraph describes the evidential uncertainty model associated with the $\hat{y_{t}}$ values. This modeling aims at discounting or softening these imputed label values according to the duration without available data before and after them. This model is therefore based on the time shifts $\delta_{t}^{p}=\left|t-p\right|$ and $\delta_{t}^{n}=\left|t-n\right|$ between the missing values and the closest known ones. The larger those time shifts, the more uncertain the corresponding imputed label $\hat{y_{t}}$. Let *β* ∈ [0, 1] be an hyper-parameter controlling the uncertainty level, *i.e.* the decreasing speed of masses $m_{t}\left(\left\{\hat{y_{t}}\right\}\right)$ in regards to the time between the missing values and the closest ones. The mass function associated with the CMA and LOCF imputation methods is:

$$\begin{array}{c} \left\{ \begin{array}{ccc} m_{t}\left(\left\{\hat{y_{t}}\right\}\right) & = & \text{exp}(-\beta \times \min(\delta_{t}^{p_{1}},\delta_{t}^{n_{1}}))\\ m_{t}\left(\Omega \right) & = & 1-m_{t}\left(\left\{\hat{y_{t}}\right\}\right) \end{array}\right. \end{array}$$ (15)

This model was tested on several experimental set-ups to study its predictive performance.

**Fig 11:**
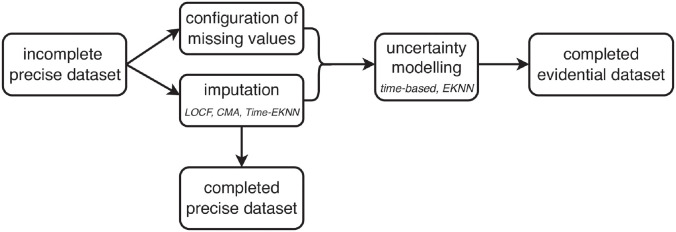
Evidential imputation scheme.

The overall articulation between imputation and associated uncertainty models is described in Fig 11.

In this section an experiment is presented on a public COVID-19 dataset in which some labels (*i.e.* daily number of deaths) are *noised*, *i.e.* randomly removed and then imputed before learning and testing phases. After describing the dataset, we give the details of our noise procedure and the experimental set-up and finally we analyze the results.

### 4.6 Dataset

**Fig 12:**
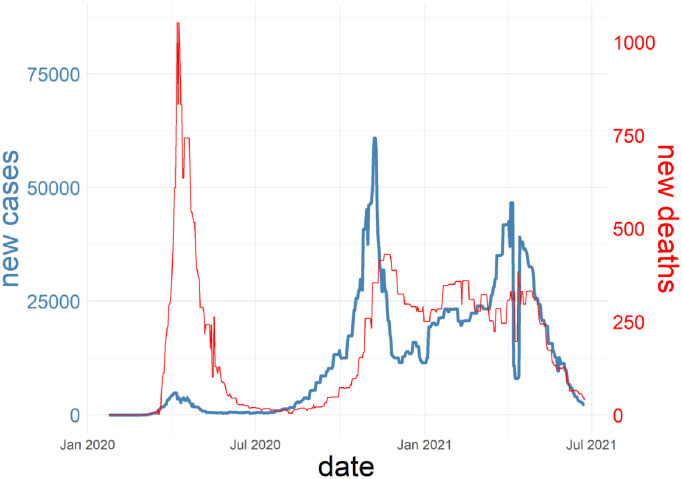
Evolution of new deaths and new cases.

On the website ourworldindata (https://ourworldindata.org/) we used the French dataset containing the number of daily confirmed new cases (*x*_*t*_)_*t*=1,…,*T*_ and new deaths (*y*_*t*_)_*t*=1,…,*T*_. Fig 12 shows the evolution of new cases and new deaths.

As the detection policy has evolved between the 2 pandemic waves, the link between new cases and new deaths seems radically different during those 2 periods. As the number of daily new cases was clearly underestimated during the first wave, we restricted the experiment to the second wave. We finally had 367 complete daily observations for this dataset.

As there are no missing values in the dataset, we randomly removed or *noised* some label values (*i.e.* new deaths). In the next subsection we give the details of our noise injection procedure.

### 4.7 Noise procedure

The proportion *v* ∈ [0, 1] of label values *y*_*t*_ to remove is the input of the procedure. In order to simulate plausible measurement errors, we removed labels *y*_*t*_ by time frame. Having randomly picked the frames centers at random, we generated reasonable frame sizes. The procedure is iterative until the proportion of removed labels reaches *v*.

**Algorithm 1:** Noise procedure for label values removing.

**Data:** original dataset, *v*: noise level

**Result:** noised dataset containing v% of missing labels

removed ← 0

*s* = ⌊*v* ⋅ *T*⌋ number of labels to remove;

**while** *removed* < *s* **do**

 frame center uniform random generation *c* ∈ [1:T];

 frame size *s* uniform random generation in ∈ {1, 2, 3};

 computation of label indices to remove {*c* − *s*, ….*c* + *s*};

 labels removal: $\left\{y_{t-s},\ldots ,y_{t+s}\right\}\to \left\{\hat{y_{t-s}},\ldots ,\hat{y_{t+s}}\right\}$;

 removed ← removed + 1 + 2*s*;

**end**

### 4.8 Smoothing

**Fig 13:**
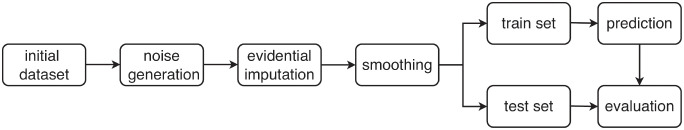
Experimental process.

Because of the weekly constraint in health policy, the raw COVID-19 data are usually *sawtooth-shaped* curves. This implies that smoothing method can and should be applied in order to get values corresponding more to the reality than the very noisy raw ones. We chose to use a moving median of 7 days (labels and features). Doing so, we avoided biases without creating unreasonable values. Since smoothed data are usually very regular, imputing missing values on smoothed data is not a real issue, we therefore decided to smooth our noised dataset after imputation. All the implemented predictive models in our study were trained and tested on smooth data because of their higher level of reliability compared to the *sawtooth-shaped* ones. The whole process is represented in Fig 13

### 4.9 Experimental set-up

In this subsection we present the chronological set-up of our experiment and the considered hyper-parameters spaces. Some hyper-parameters have been set *a priori*: *R* and *U* representing the width of the CMA approach (12) were both fixed at 5 days. As hospital reorganization involves strong administrative constraints incompatible with too short or too large horizon, the considered prediction horizon *h* was 7 days. Finally, the uncertainty hyper-parameter of the time-based uncertainty model (15) *β* was set at 0.05.

For the other hyper-parameters, several configurations were considered:

- noise level: *v* ∈ {0, 0.1, …, 0.7}
- data historical length: *q* ∈ {1, 2, …, 7}
- number of neighbors for the E*K*NN regressors: *K* ∈ {1, 10, 20}

The data historical length *q* represents the number of past data (deaths and cases) representing each training example. The first 21 dates are set aside for training, the predictions *y*_*t*_ are then computed iteratively at each date *t* from all the past couples data (*x*_*t*^′^_, *y*_*t*^′^_)_*t*^′^=*t*−*h*,…,*t*−(*h*+*q*)_. At first iteration of the chronological evaluation, the 21 first days are used as training data in order to predict the label value of the 21 + 7 = 28^*th*^ day (with a prediction horizon of *h* = 7 days). After that, the training data are augmented by one date at each iteration, for example at the second iteration we use the 22 first days to predict the label values of the 29^*th*^ day.

Each complete chronological evaluation is repeated 50 times because of the randomness of the noise procedure and predictions are averaged.

$$\begin{array}{c} {\left(\Omega_{Y}\right)}_{t}=\left\{0,1,\ldots ,\underset{t^{'}<t}{\text{max}}\left(y_{t^{'}}\right)\times 1.15\right\} \end{array}$$ (16)

Since Ω_*Y*_ must be defined before the prediction step in the regression of the E*K*NN we propose (see Eqs (8), (9) and (10)), we redefine it at each iteration according to Eq 16 by updating the maximum label value in the training data. We chose a safety margin of 15% in regards to the real maximum number of deaths observed. As a baseline we considered the moving average that predicts the number of deaths for the next week as the average of the previous 2 weeks.

Two types of figures are presented. The chronological evaluations (see Figs 2 and 3) allow us to visually evaluate the predictions of the regression for different noise levels according to different imputation methods by comparing the predicted labels with the real ones. E*K*NN regression models are evaluated without and with uncertain models (“E*K*NN” and “E*K*NN uncertain labels”). In the former case, imputed training labels are considered certain whereas in the latter case uncertain models (see Eqs (15) and (8)) allow the E*K*NN regression to take into account data imputation uncertainty.

The noise level sensitivity of the complete evaluations is represented in Figs 6 and 5 where the predictive performance is measured according to the noise level. The evaluation metric we considered is the Root Median Squared Error (RMedSE). We chose it rather than the standard Root Mean Square Error (RMSE) because of the high sensitivity of the mean operator to extreme values which are quite usual in the COVID-19 data.

We evaluate the imputation methods by comparing the initial dataset with the imputed ones in terms of RMSE in order to evaluate the imputation errors that were unlikely to contain extreme values (see Fig 1).
